# Role of Zinc Homeostasis in the Pathogenesis of Diabetes and Obesity

**DOI:** 10.3390/ijms19020476

**Published:** 2018-02-06

**Authors:** Ayako Fukunaka, Yoshio Fujitani

**Affiliations:** Laboratory of Developmental Biology & Metabolism, Institute for Molecular & Cellular Regulation, Gunma University, 3-39-15 Showa-machi, Maebashi, Gunma 371-8512, Japan

**Keywords:** zinc, zinc transporters, diabetes, obesity, ZnT8, ZIP13, pancreatic β cell, beige adipocyte, therapeutic target

## Abstract

Zinc deficiency is a risk factor for obesity and diabetes. However, until recently, the underlying molecular mechanisms remained unclear. The breakthrough discovery that the common polymorphism in zinc transporter *SLC30A8*/ZnT8 may increase susceptibility to type 2 diabetes provided novel insights into the role of zinc in diabetes. Our group and others showed that altered ZnT8 function may be involved in the pathogenesis of type 2 diabetes, indicating that the precise control of zinc homeostasis is crucial for maintaining health and preventing various diseases, including lifestyle-associated diseases. Recently, the role of the zinc transporter ZIP13 in the regulation of beige adipocyte biogenesis was clarified, which indicated zinc homeostasis regulation as a possible therapeutic target for obesity and metabolic syndrome. Here we review advances in the role of zinc homeostasis in the pathophysiology of diabetes, and propose that inadequate zinc distribution may affect the onset of diabetes and metabolic diseases by regulating various critical biological events.

## 1. Introduction

Type 2 diabetes is now a crucial health problem in many parts of the world. Type 2 diabetes mellitus (T2DM) is characterized by peripheral insulin resistance and pancreatic beta (β) cell dysfunction. The disease is thought to be caused by defects in insulin signaling or secretion, the activation of various stress pathways, and dysregulation of the central nervous system (CNS). It is well accepted that the most accurate predictor for developing T2DM is obesity. Therefore, much attention has also been paid to the contribution of nutrients and nutrient-sensing pathways in situations of chronic caloric excess. Most of the interest in the role of nutrients in diabetes is centered on macronutrients, such as carbohydrate and fat, but micronutrients, such as iron and zinc, are also closely associated with diabetes [1,2]. Whole-body level dysregulation of zinc is known to occur in both type 1 and type 2 diabetes. However, it remains unclear as to whether zinc deficiency causes the disease or is merely a consequence of the disease. A possible causal link between changes in zinc homeostasis and pancreatic β cell function was suggested in 2007 with the identification of an association between the risk of T2DM and polymorphisms in the *SLC30A8* gene, which encodes zinc transporter ZnT8 [3].

Several groups have been analyzing the roles of zinc homeostasis in the health and disease of endocrine organs, with particular focus on zinc transporter function. In this review, we will discuss the roles of zinc homeostasis in glucose metabolism, particularly in association with ZnT8. Furthermore, we will discuss the role of ZIP13 in beige adipocyte biogenesis and energy expenditure, which we have recently elucidated.

## 2. Zinc Homeostasis and Pancreatic β Cells

### 2.1. Insulin Biosynthesis in Pancreatic β Cells

Pancreatic β cells are known to contain very high concentrations of zinc compared with various other cells. In particular, insulin secretory granules have been shown to have the highest zinc content within β cells [4,5]. In vertebrates, three protein families have been shown to regulate cellular zinc homeostasis, namely, metallothioneins (MTs), zinc importers (ZIP, *SLC39A*), and zinc exporters (ZnT, *SLC30A*). MTs have been shown to bind zinc with low affinity, whereas zinc transporters mediate the compartmentalization of zinc into various organelles and vesicles for their storage, and to supply zinc to various proteins that require zinc for their function [6]. Nine ZnTs and 14 ZIP transporters have been identified to play important roles in whole body maintenance, as well as in zinc homeostasis at the cellular and subcellular levels. These transporters act either independently or coordinately, and in a cell-specific or tissue-specific manner [7,8]. ZnT8 plays a key role in the accumulation of zinc within insulin secretory granules [9]. Furthermore, zinc is essential for the appropriate synthesis of insulin, as well as its storage and structural stability [10].

Insulin comprises a hexamer of six insulin and two zinc molecules [11,12]. The mature insulin molecule comprises two polypeptide chains, namely, chains A and B. Initially, insulin mRNA is translated into an inactive preproinsulin molecule, which comprises two chains that are connected by a c-peptide, with the signal peptide at the N-terminus. Proinsulin is formed from preproinsulin by signal peptide cleavage in the endoplasmic reticulum (ER). Proinsulin then folds into the final three-dimensional structure, upon formation of the correct disulfide bonds. Subsequently, the proinsulin protein forms dimers via electrostatic interactions. Proinsulin hexamers are then formed by electrostatically-coupled proinsulin dimers and zinc binding to histidine residue 10 of the B chain (His B10) [13]. After entering the Golgi apparatus, insulin hexamer formation is completed upon the dissociation of c-peptide, mediated by prohormone convertase (PC) dissociation [14]. Insulin crystallization occurs under specific conditions in insulin secretory granules, in which both insulin and zinc exist in high concentrations and acidic pH is maintained [15,16]. This crystallized insulin can be observed as “dense core granules” by electron microscopy [17,18], and insulin crystals that are secreted from pancreatic β cells are believed to dissociate rapidly into monomers as they enter the bloodstream.

### 2.2. Zinc Supplementation in Diabetic Animals and Patients

Mice with zinc deficiency were found to have a decreased number of insulin granules in their pancreatic β cells [19], as well as impaired glucose-stimulated insulin secretion (GSIS) [20]. Since pancreatic β cells synthesize a large amount of ATP, this makes them prone to oxidative stress exposure, which can subsequently cause cellular damage [21]. As zinc is required for the actions of many antioxidative enzymes, including Cu-Zn-SOD (superoxide dismutase) [22] and catalase [23], a lack of zinc will lead to further damage of pancreatic β cells under oxidative stress, such as in T2DM.

Regarding humans, a prospective cohort study in the United States analyzed 82,000 women and demonstrated that low zinc intake results in a 17% increased risk of developing diabetes compared with those women taking sufficient amounts of zinc [24]. Recently, a study from China has reported a negative correlation between concentrations of plasma zinc and the onset of diabetes [25]. Interestingly, this report suggested that an interaction between SLC30A8 (ZnT8) dysfunction and decreased plasma zinc concentrations regulates glucose tolerance and diabetes. Furthermore, the authors suggested that a decrease in plasma zinc concentrations as well as ZnT8 function may coordinately increase the risk of diabetes. Although these data suggest that zinc supplementation prevents disruption of glucose homeostasis, particularly in people with zinc deficiency, prospective intervention studies should be performed to clarify the efficacy of zinc supplementation in preventing the onset of diabetes. On the other hand, excessive supplementation with zinc may have deleterious effects, as excessive zinc intake may cause an undesirable increase in HbA1c levels and high blood pressure [26].

A genome-wide association study (GWAS) demonstrated that a nonsynonymous single-nucleotide polymorphism, namely, rs13266634 in the *SLC30A8* gene, results in the replacement of tryptophan-325 to arginine, which modestly increases the risk of T2DM [3]. Furthermore, recent studies on human SNPs demonstrated the association of 12 rare loss-of-function ZnT8 mutants with a 65% decreased risk of T2DM [27]. Taken together, the data indicate that polymorphisms in the *SLC30A8* gene are associated with altered risk of T2DM.

## 3. ZnT8 Plays a Crucial Role in Glucose Homeostasis

### 3.1. Insulin Secretory Granule of ZnT8-KO Mice

ZnT8 is found in the plasma membrane of insulin secretory granules of pancreatic β cells, and is implicated in zinc transport into insulin secretory granules [9]. Several groups, including our own have aimed to clarify the role of ZnT8 in glucose homeostasis by establishing *SLC30A8*-deficient (ZnT8-KO) mice [18,28,29] (Supplemental Table S1). Each mouse model shows variation in certain phenotype traits, which are attributed to differences in deletion strategy, genetic background, and housing condition. Most of the ZnT8-KO mice have been reported to have mildly impaired glucose tolerance and there have been no reports of the improvement of glucose tolerance in the ZnT8-KO mice model [18,30,31,32,33,34,35] (Supplemental Table S1). Furthermore, hZnT8 transgenic mice showed mildly improved glucose tolerance [31], suggesting that the expression levels of ZnT8 determines the risk of T2DM in these mouse models. Electron microscopy analysis revealed that dense-core granules, which are a hallmark of crystallized insulin usually seen in normal β cells, were absent in the ZnT8-KO mouse β cells. In most of the ZnT8-KO mice, some of the granules appeared atypical granules possessing abnormal “rod-like” or empty cores, while some ZnT8-KO mice revealed the nearly complete loss of crystal containing granules [18,30].

### 3.2. Phenotypes of ZnT8-KO Mice Regarding Glucose Metabolism

As described above, glucose tolerance was mildly impaired in ZnT8-KO mice. This demonstrates that peripheral insulin levels in ZnT8-KO mice were reduced compared with control mice. Indeed, we and others have observed decreased insulin levels in ZnT8-KO mice, despite GSIS levels being unchanged or slightly increased in ZnT8-KO mouse islets [18,34] (Supplemental Table S1). To understand this discrepancy, we performed a pancreas perfusion experiment, and found that insulin secretion was still enhanced upon pancreas infusion in ZnT8-KO mice, further supporting that insulin secretion is increased in ZnT8-KO mice. However, pancreas-liver dual perfusion analysis demonstrated that in these mice, a large proportion of the secreted insulin is actually degraded during its passage through the liver, suggesting that ZnT8 regulates hepatic insulin clearance [18]. A set of in vivo and in vitro experiments demonstrated that ZnT8-mediated zinc inhibits hepatic insulin uptake by counteracting clathrin-mediated endocytosis of the insulin receptor. These results suggested that ZnT8 plays an important role in determining the amount of insulin that is delivered to the liver and other peripheral organs, and thus optimizes the effect of insulin on whole body glucose metabolism [18].

**Figure 1:**
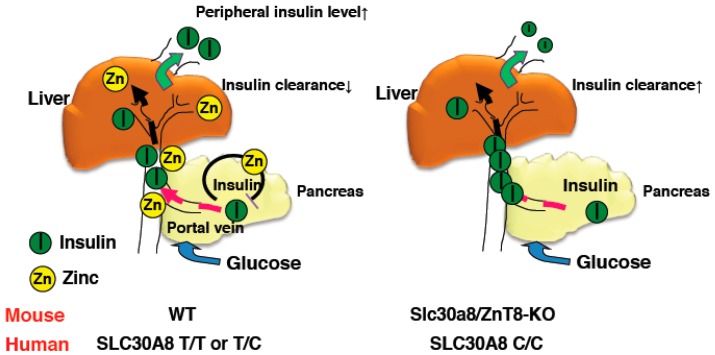
Schematic representation of insulin clearance in WT and ZnT8-KO mice. Zinc co-secreted with insulin suppresses insulin secretion from pancreatic β cells and inhibits hepatic insulin clearance in WT mice (**left**). In contrast, reduced zinc secretion results in enhanced insulin secretion from β cells in ZnT8-KO mice and hepatic insulin clearance is not suppressed (**right**). Thus, peripheral insulin levels in ZnT8-KO mice are maintained at lower levels than in WT mice.

For the assessment of in vivo insulin clearance, the c-peptide/insulin ratio may be useful [36,37]. Consistently, although insulin secretion was increased in ZnT8-KO mice, peripheral insulin levels were lower and c-peptide/insulin ratios were increased [18]. Studies of c-peptide/insulin ratios and rates of insulin clearance in humans with the rs13266634 polymorphism also demonstrated results consistent with this idea [18]. Furthermore, the Eugene study showed that when human homozygous carriers of the *SLC30A8* risk allele are subjected to the intravenous glucose tolerance test, they demonstrate low peripheral insulin levels in the early phases [38]. These results indicate that SLC30A8/ZnT8 regulates hepatic insulin clearance, and importantly, the same mechanism appears to be conserved in humans (Figure 1) [18].

Clinically, the inhibition of hepatic insulin clearance seems likely to be a therapeutic target for diabetes. A previous study reported the therapeutic potential of small molecule inhibitors of insulin-degrading enzyme, which regulates insulin catabolism [39,40]. Our findings hence might provide novel insights into the molecular pathology of diabetes, which involves dysregulated insulin clearance from the liver, and hence may be a promising future therapeutic target for diabetes [18].

### 3.3. Involvement of Other ZnT Transporters

Although there is a substantial decrease in total zinc levels in the islets of ZnT8-KO mice compared with wild-type mice, the phenotypes of ZnT8-KO mice regarding glucose metabolism were fairly modest. Several other ZnT isoforms were expressed at low levels in the pancreatic islets. Thus, functional compensation by other ZnT isoforms might reduce the effect of the ZnT8-KO phenotype. ZnT3 is a candidate ZnT transporter for this compensation. ZnT3 is known to play a role in the uptake of zinc in the synaptic vesicles of glutaminergic hippocampal neurons [41,42]. Considering that β cells and neurons share some similar characteristics, ZnT3 might be involved in the transport of zinc into insulin secretory vesicles. However, it is unclear whether ZnT3 is expressed in the islets of mice [43], and ZnT3-KO mice appear to undergo normal glucose metabolism [44], suggesting that ZnT3 is not involved in this process.

As zinc is required for the hexamerization of insulin and its conversion from proinsulin to insulin in the Golgi compartment, a sufficient amount of import of zinc to this compartment is also required. ZnT5 and ZnT7 are also reported to be expressed in β cells and to co-localize with the Golgi apparatus and secretory vesicles [43,45,46]. Thus, ZnT5 and ZnT7 transporters might be involved in these processes. A recent study analyzed this possibility by crossing ZnT7-KO mice with ZnT8-KO mice. However, whether ZnT7 has a redundant role of ZnT8 remains to be clarified because global ZnT7-KO mice displayed several defects in insulin-sensitive tissues outside of β cells, as described below, and because the report did not include data on ZnT8 single-knockout mice [47]. Further analyses are needed to identify the zinc transporters involved in each step from insulin processing to storage.

### 3.4. Zinc Transport Activity of ZnT8 Variants

**Figure 2:**
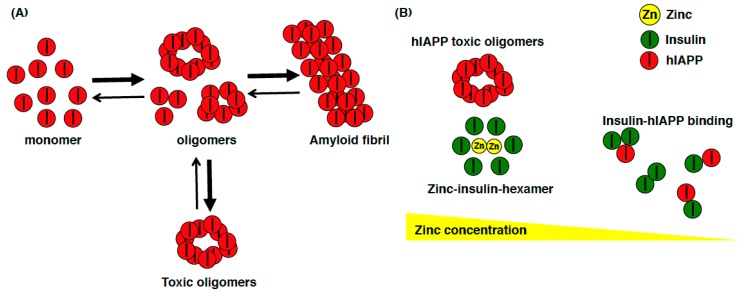
Schematic model of the relationship among hIAPP, insulin, and zinc. (**A**) hIAPP can easily form toxic oligomers that induce apoptosis and amyloidogenesis in β cells in T2DM patients; (**B**) When the zinc concentration in insulin secretory granules is high, zinc is used to form zinc-insulin-hexamer, and hIAPP can easily form toxic oligomers. On the other hand, when the zinc concentration is low in insulin secretory granules (such as insulin secretory granule in ZnT8-KO mice), insulin exists as monomer or dimer, which preferentially binds to hIAPP monomer and prevents hIAPP from self-associating and aggregating.

One of the important unresolved issue regarding ZnT8 is whether the ZnT8 variant 325Arg(R) increases or decreases zinc transport activity. In one study, the fluorescent dye FluoZin-3 was used to monitor cytosolic zinc and the fluorescent dye Zinquin was used to monitor vacuolar zinc accumulation in MIN6 cells transiently expressing the Arg(R) or Trp(W) variants of hZnT8. Cells expressing the variant (W) showed significantly greater fluorescence of both dyes, and the authors concluded that this variant was a more active transporter of zinc [34]. In another study, HEK293 cells inducibly expressing hZnT8 variants were established, and both the R and W variants of hZnT8 were purified in the native state, and a reconstitution system was developed to measure zinc transport activities [48]. The authors found the R variant to be more active than the W variant, suggesting that the common high-risk R variant is hyperactive and thus may be a therapeutic target to reduce the risk of T2DM in the general population [48]. An additional report described the possible association between the diabetes risk allele in hZnT8 and the higher zinc concentration in human islets [49]. These results suggest that β cells with lower zinc levels may be protection from T2DM, whereas higher zinc in β cells may be associated with T2DM. Interestingly, these new findings appear to be consistent with the finding that rare loss-of-function mutations in *ZnT8* are associated with reduced T2DM risk in humans [27]. This suggests that the role of ZnT8 might be contradictory between humans and mice, as a loss-of-function of ZnT8 in humans decreases the risk for T2DM, whereas ZnT8-KO mice have impaired glucose tolerance [50]. In the course of evolution, the role of ZnT8 in glucose homeostasis has been altered. There are some factors to explain this discrepancy. Since synaptic ZnT3-mediated zinc contributes predominantly to amyloid deposition in human amyloid precursor protein (hAPP) mice [51,52], human islet amyloid polypeptide (hIAPP) might be able to explain this discrepancy. Compared to mouse IAPP, hIAPP can form toxic oligomers, which affect β cells by inducing apoptosis and amyloidogenesis in T2DM [53,54] (Figure 2A). A recent computational analysis showed that zinc concentration determines insulin oligomer equilibrium and that the hIAPP monomer preferentially binds to both the insulin monomer and dimer, compared to the formation of hIAPP homodimer. Therefore, regarding the loss of ZnT8 function, the zinc deficiency shifts the equilibrium of the insulin oligomers toward monomers and dimers, which isolate hIAPP monomers (nontoxic form) and prevent hIAPP from self-association and subsequent aggregation (toxic forms), thereby reducing the risk of T2DM (Figure 2B) [55]. The theory of altered hIAPP aggregation in β cells in response to altered ZnT8 function is a promising but still correlative hypothesis at this point. Therefore, this hypothesis can be validated by creating hIAPP transgenic (hIAPP-Tg) mice from ZnT8-KO mice, to investigate whether hIAPP cytotoxicity can be ameliorated by the deletion of ZnT8.

## 4. Zinc Distribution Affects Adipocyte Metabolism

### 4.1. Zinc Distribution in Obesity

Chronic low intake of zinc is associated with an increased risk of diabetes. Hence, zinc supplementation is expected to be an effective method for preventing metabolic syndrome and diabetes. A previous study analyzed the effect of zinc supplementation to prepubertal obese children on insulin resistance and metabolic syndrome. Zinc supplementation was suggested to be a useful and safe additional intervention treatment [56]. However, to our knowledge, the effectiveness of zinc supplementation for the treatment of obesity and diabetes has not been demonstrated in large-scale studies, particularly in adults. Therefore, it is important to establish the safety, efficacy, and effective dose of zinc supplementation in adults.

**Figure 3:**
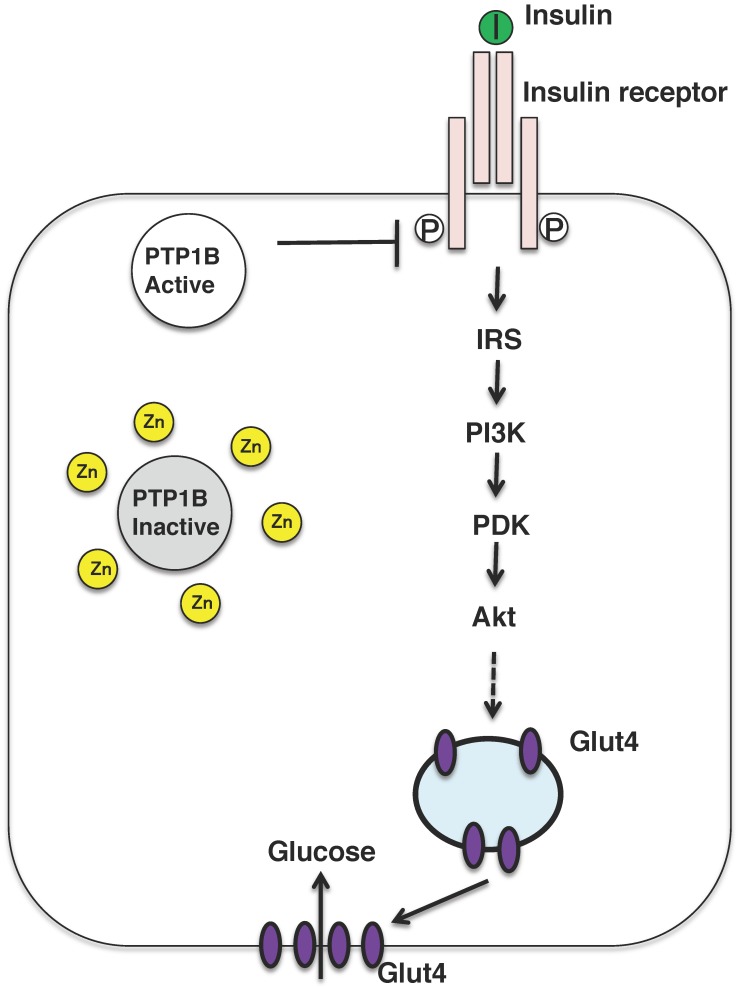
Insulin signaling pathway and insulin mimicking function of zinc ions. Insulin binds to the insulin receptor located in the plasma membrane in the peripheral tissues, such as liver and muscle. The insulin-signaling pathway is activated and the glucose transporter GLUT4 is translocated to the plasma membrane. Zinc might inhibit the activity of PTP1B, which activates the insulin-signaling pathway. PI3K, phosphatidylinositol-3-kinase; IRS, insulin receptor substrate; PKD, protein kinase D.

Nevertheless, zinc might be referred to as the insulin-mimetic, since zinc stimulates lipogenesis and glucose uptake in isolated adipocytes, and zinc ion acts as an insulin-mimetics through their direct effect on the insulin-signaling pathway [2]. The insulin-sensitizing effect of zinc has been attributed to the inhibition of the tyrosine phosphatase activity of protein tyrosine phosphatase 1B (PTP1B) (Figure 3) [57,58]. Zinc ion inactivates PTP1B by non-covalent binding to its cysteine residues which is crucial for the enzymatic activity and reactive oxygen species is also known to inactivate the enzyme in a similar manner. In fact, oxidative stress is upregulated in the most of the patients with diabetes and diabetic animals, further mechanistic analysis is needed to examine the risks and benefits of zinc supplementation as a mean for mitigating obesity and type 2 diabetes.

### 4.2. Association between Adipocyte Metabolism and Zinc Homeostasis

Obesity and its associated metabolic diseases develop when energy intake exceeds energy expenditure; this can be caused by decreased physical activity, the inability of the CNS to downregulate appetite, or the ingestion of high-calorie foods [59]. Adipose tissue is involved in energy storage and also functions as an endocrine organ to release free fatty acids (FFA) and adipokines, such as leptin, tumor necrosis factor-alpha (TNF-α), interleukin-6 (IL-6), and adiponectin [60]. In addition, adipose tissue comprises numerous types of stromal cells, including preadipocytes, endothelial cells, immune cells, and fibroblasts. During the course of obesity, adipocyte cells and stromal cells in adipose tissue change in number and characteristics. Invasion of macrophages into the adipose tissue of obese individuals is associated with increased secretion of inflammatory adipocytokines, including TNF-α and IL-6, which leads to insulin resistance [61]. Macrophages have recently been reported to be involved in adipose tissue inflammation as well as in the regulation of adipose metabolism through the disrupted modulation of adipocytokines production [61].

Several reports have addressed the association between zinc transporters and adipose metabolism. For example, in the adipose tissue of *Zip14*-KO mice, hypertrophy together with enhanced proinflammatory signaling is observed through activation of nuclear factor-kappaB (NF-κB) and the Janus-activating kinase 2 (JAK2)/signal transducer and activator of transcription 3 (STAT3) pathway, and this might contribute to obesity-induced insulin resistance [62]. Consistent with this idea, *Zip14* expression is significantly reduced in obese individuals compared with non-obese individuals, and is increased markedly following weight loss [63].

Adipose tissue plays a crucial role in controlling energy balance. It comprises white and brown adipocytes, which perform different functions. White adipocytes store excess energy, whereas brown adipocytes play a role in energy expenditure [59]. In mammals, brown adipose tissue (BAT) dissipates energy in the form of heat and acts as a defense mechanism against hypothermia. Brown adipocytes are unique in that they have a very large number of mitochondria and are also able to metabolize glucose and fats to produce heat rather than ATP. This thermogenic activity of brown adipocytes is mediated largely via the actions of uncoupling protein-1 (UCP1) [64]. Furthermore, two distinct types of thermogenic adipocytes have recently been identified, namely, the “classical brown adipocytes” and the “beige adipocytes”. Beige adipocytes are induced in white adipose tissue (WAT), particularly in inguinal WAT upon various external factors, including exercise, chronic cold exposure, and bariatric surgery [65]. Identification and implementation of therapies based on beige fat require a detailed understanding of the differences in the developmental mechanisms and functions of white, brown, and beige adipocytes.

**Figure 4:**
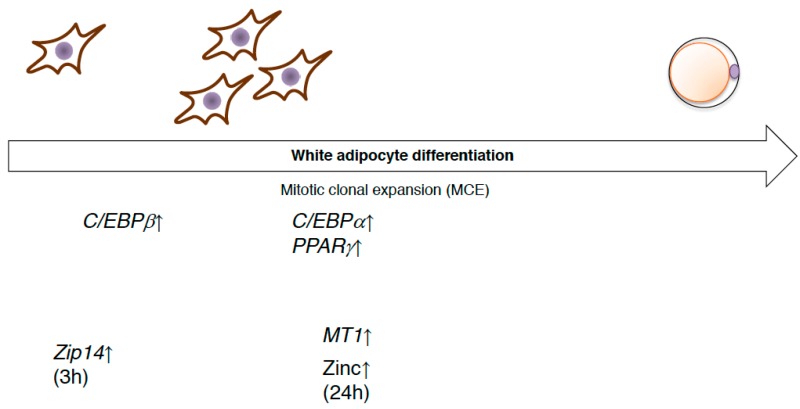
The expression of zinc transporters during white adipocyte differentiation. Preadipocytes are differentiated and trigger DNA replication and reentry into the cell cycle (mitotic clonal expansion, MCE). The expression of several genes related to zinc homeostasis is altered.

Many transcriptional regulators and transcription factors are used for differentiation into these various fat cell types, such as peroxisome proliferator-activated receptor gamma (PPARγ) and the CCAAT/enhancer binding protein (C/EBP) family of transcription factors, respectively [66]. The induced differentiation of preadipocytes triggers DNA replication and reentry into the cell cycle (mitotic clonal expansion). Mitotic clonal expansion involves a transcription factor cascade, followed by the expression of adipocyte genes. A critical event is phosphorylation of C/EBP-β, which is a form of activated C/EBP-β, which then triggers PPARγ and C/EBP-α, which in turn coordinately activate genes whose expression produces the adipocyte phenotype, such as *aP2* [67]. Several studies have analyzed the roles of zinc and the zinc transporter in the differentiation process of white adipocytes. Expression of the *Zip14* gene was found to be upregulated during early adipocyte differentiation [63,68]. During mitotic cell expansion, zinc and *MT* levels within cells are rapidly increased. This elevation is essential for the transition from G0/G1- to S-phase of the cell cycle [69] (Figure 4). ZnT7-KO mice show a mild zinc deficiency, with low body weight gain as well as body fat accumulation. The underlying mechanism of these characteristics in ZnT7-KO mice is that ZnT7 is likely to be involved in lipogenesis in adipocytes, rather than in the early adipocyte differentiation process, such as mitotic clonal expansion [70].

Most of the transcription factors that are known to direct cells toward a brown/beige adipocyte lineage instead of a white adipocyte lineage act via the core transcriptional machinery of adipogenesis. PR domain containing 16 (PRDM16), which is an essential transcriptional coregulators of brown/beige adipocyte differentiation [71], determines the brown/beige adipocyte lineage mainly via its interaction with various transcriptional factors, including PPARγ, peroxisome proliferator-activated receptor γ coactivator 1-α (PGC-1α), C/EBP-β, and zinc finger protein 516 [65,72]. Among them, many zinc-containing transcriptional factors participate in brown/beige adipocyte differentiation and function.

### 4.3. ZIP13 Regulates Beige Adipocyte Biogenesis and Energy Expenditure

As explained above, we have been investigating the roles of zinc homeostasis in the health and disease of endocrine organs by focusing on the biological functions of zinc transporters. *Zip13*-KO mice were reported to show impaired bone formation and growth retardation [73]. Importantly, ZIP13 is also plays a crucial role in connective tissue development in humans. Patients with a loss-of-function mutation in ZIP13 showed a similar phenotype to *Zip13*-KO mice and were diagnosed as having a novel type of Ehlers-Danlos syndrome. Interestingly, human patients of Ehlers-Danlos syndrome were reported to display lipoatrophy [73]. Therefore, we aimed to clarify the roles of ZIP13 in fat tissue. During our investigation, we found that *Zip13*-KO inguinal WAT had a high number of functional beige adipocytes [74]. Furthermore, *Zip13*-KO mice showed a significantly higher oxygen consumption rate than wild-type mice, although there were no differences in food intake, suggesting that *Zip13*-KO mice have a tendency to not gain weight. Consistent with this idea, *Zip13*-KO mice showed resistance to high fat diet-induced obesity [74].

**Figure 5:**
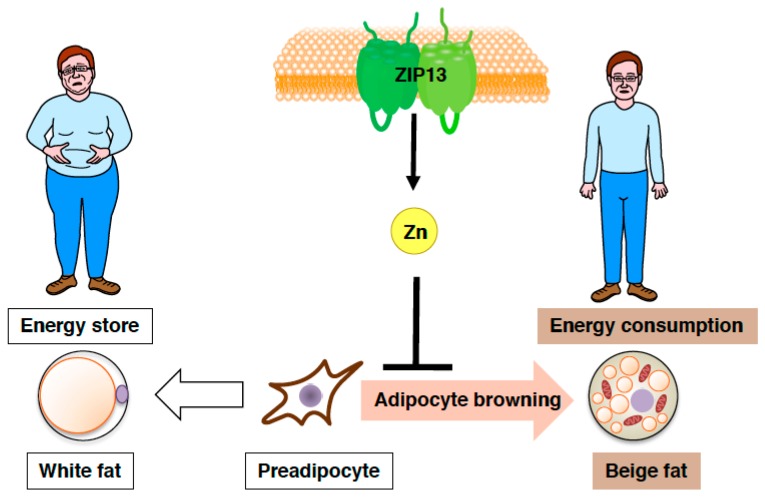
Zinc transporter ZIP13 inhibits adipocyte browning. Schematic model of the role of ZIP13 in adipocyte browning.

Furthermore, both gain-of-function and loss-of-function experiments have demonstrated that the accumulation of C/EBP-β, which is involved in determining brown/beige adipocyte lineage in cooperation with the dominant transcriptional coregulator PRDM16, is crucial for the increased adipocyte browning resulting from the loss of ZIP13 [74], implying most likely that ZIP13-mediated zinc transport is required for the inhibition of adipocyte browning (Figure 5), and that ZIP13 may deliver zinc ions to specific molecular targets that control the function of C/EBP-β or/and other target proteins. Further analyses of *Zip13*-deficient cells will clarify the specific roles of ZIP13 in beige adipocyte biogenesis.

Importantly, the presence and activity of thermogenic beige adipocytes are associated with improved global metabolic fitness, such as improvements in insulin resistance and glucose homeostasis [65]. Indeed, *Zip13*-KO mice reportedly have improved glucose tolerance and insulin tolerance compared with control mice in addition to increased energy expenditure [74]. Furthermore, given that recent studies demonstrated that adult human brown adipocytes share biological characteristics with rodent beige adipocytes, rather than rodent brown adipocyte [75]. The results of our study may hence contribute to the establishment of novel treatments for obese diabetic patients via the manipulation of adipocyte identity.

## 5. Involvement of Zinc in the Insulin Signaling Pathway

### 5.1. Zinc Transporters Affect Skeletal Muscle Insulin Signaling

Skeletal muscle plays a key role in insulin-stimulated glucose uptake in the postprandial state. Under insulin-resistant conditions, there is a decrease in insulin signaling via IRS-1, PI3K, and Akt, resulting in decreased translocation of the GLUT4 glucose transporter, to the plasma membrane, as well as decreased insulin-stimulated glucose transport into cells (Figure 3).

Skeletal muscle is the major reservoir for zinc, containing approximately 60% of the total whole-body zinc [76]. Zinc transporter SLC39A7 (ZIP7) has been reported to be involved in glycemic control within skeletal muscle. The knockdown of *Zip7* in the mouse skeletal muscle cell line C2C12 demonstrated that glucose metabolism is enhanced by ZIP7 via Akt phosphorylation, in which ZIP7-mediated zinc activates insulin receptor signaling via its binding to PTP1B [77]. A different study reported that ZnT7-KO mice demonstrate impaired glucose tolerance and insulin sensitivity, resulting from a decrease in the insulin signaling pathway, such as decreased levels of Akt phosphorylation in skeletal muscle and adipocytes, as described above, which thereby reduce glucose uptake [78]. Although many studies have been performed to analyze the insulin-mimetic actions of zinc [79], the specific zinc transporters that are responsible for initiating these signaling processes remain unclear. In particular, given that ZIP7 acts as the gatekeeper of zinc release from the Golgi apparatus [80] and that insulin-signaling cascades in skeletal muscle are affected by ZIP7-mediated zinc, it will be very important to analyze the metabolic phenotypes of skeletal muscle in tissue-specific *Zip7*-KO mice.

### 5.2. Zinc Homeostasis and Sarcopenia

Sarcopenia is the age-associated degenerative loss of skeletal muscle mass, quality, and strength. In older people, sarcopenia is often accompanied by diabetes [81]. Some of the mechanisms involved in the development of sarcopenia, such as insulin resistance, mitochondrial dysfunction, and chronic inflammation, are also thought to play roles in the pathogenesis of diabetes [82]. Recently, the zinc/zinc transporter and *MT* expression levels have been reported to be upregulated during skeletal muscle atrophy. For example, the blocking of MTs 1 and 2 has been shown to increase skeletal muscle mass and strength [83], suggesting the potential of MTs as therapeutic targets for sarcopenia. Furthermore, muscle zinc levels and *Zip14* expression levels are known to increase with age [84], although the underlying mechanisms and physiological meaning of these observations remain to be clarified. Further analyses are required to understand the precise roles of zinc transporters in sarcopenia that occurs in association with diabetes.

## 6. Summary

Since human patients with *Zip13* deficiency have been reported to show lipoatrophy, we expected *Zip13*-KO mice to be resistant to insulin [73]. However, on the contrary, *Zip13*-KO mice showed improved insulin sensitivity owing to the acceleration of adipocyte browning. To our knowledge, our data are the first to show that the Golgi-to-cytoplasm transport of zinc by ZIP13 is necessary for the regulation of beige adipocyte metabolism and insulin sensitivity, because simple zinc supplementation cannot inhibit adipocyte browning. On the other hand, a study of ZnT8-KO mice also showed that the release of adequate amounts of zinc from β cells together with insulin is important for the regulation of insulin clearance, suggesting that the zinc transported by ZnT8 acts as a signaling molecule between organs and mediates pancreas-to-liver organ communication. ZnT7-KO mice demonstrate mild zinc deficiency accompanied with low body weights and very little accumulation of body fat. Dietary zinc supplementation in ZnT7-KO mice cannot rescue these phenotypes. The main tissue responsible for the phenotypes of ZnT7-KO mice might be adipose tissue, which results in impaired glucose tolerance and insulin sensitivity.

Considering the aforementioned phenotypes of zinc transporter KO mice, we would like to suggest a new perspective; that adequate local zinc delivery by zinc transporters are important, and their disruption leads to the pathogenesis of a variety of diseases. In particular, as sarcopenia is an important issue in aging societies, zinc transporters or factors associated with zinc homeostasis might be candidate biomarkers or therapeutic targets. Further analyses are required toward the development of zinc transporter-mediated therapies against both obesity and diabetes.

## Supplementary Materials

Supplementary materials can be found at http://www.mdpi.com/1422-0067/19/2/476/s1.
