# Supplementary material for: Role of Zinc Homeostasis in the Pathogenesis of Diabetes and Obesity
Source: Int J Mol Sci. 2018 Feb 6;19(2):476. doi: 10.3390/ijms19020476 (PMC5855698; doi:10.3390/ijms19020476)
Supplement: Supplementary file 1 [file ijms-19-00476-s001.pdf]

Table 1. Summary of ZnT8-KO mouse studies

Study	Deletion location	Deletion strategy	Genetic background	Crystalization of insulin granules	Proinsulin concentration	Glucose intolerance	Glucose-stimulated insulin secretion	Insulin level in vivo
Nicolson et al. (2009)	Whole body	Exon 1	Mixed	granule/ rod-shaped cores	Unchanged	Impaired (only males)	Increased	Impaired
Pound et al. (2009)	Whole body	Exon 3	Mixed	Not examined	Not examined	Normal	Decreased	Impaired
Lemaire et al. (2009)	Whole body	Exon 1	Mixed	Loss of dense core granule	Unchanged	Normal	Unchanged	Unchanged
Wijesekara et al. (2010)	specific (Ins2Cre)	Exon 1	Mixed	granule/ rod-shaped cores	Increased	Impaired	Decreased	Unchanged
Pound et al. (2012)	Whole body	Exon 3	C57BL/6J	Unchanged	Decreased	Impaired	Decreased	Unchanged
Tamaki et al. (2013)	specific (Ins2Cre)	Exon 5	C57BL/6J	Loss of dense core granules	Increased	Impaired	Increased	Impaired
Mitchell et al. (2016)	specific (Ins1Cre)	Exon 1	C57BL/6J	granule/ rod-shaped cores	Not examined	Impaired	Unchanged	Impaired
